# Survey of both hepatitis B virus (HBsAg) and hepatitis C virus (HCV-Ab) coinfection among HIV positive patients

**DOI:** 10.1186/1743-422X-6-202

**Published:** 2009-11-18

**Authors:** Mohsen Mohammadi, Gholamreza Talei, Ali Sheikhian, Farzad Ebrahimzade, Yadollah Pournia, Ehsan Ghasemi, Hadis Boroun

**Affiliations:** 1 Department of Microbiology School of Medicine, Lorestan University of Medical Sciences, Khoram Abad , Iran; 2 Departments of Immunology School of Medicine, Lorestan of University Medical Sciences, Khoram Abad, Iran; 3 Department of Biostatistics, School of Medicine, Lorestan University of Medical Sciences Khoram Abad, Iran; 4 School of Medicine, Lorestan University of Medical Sciences Khoram Abad, Iran; 5 Department of Microbiology School of Medicine, Ilam University of Medical Sciences Ilam, Iran

## Abstract

**Background:**

HIV, HBVand HCV is major public health concerns. Because of shared routes of transmission, HIV-HCV coinfection and HIV-HBV coinfection are common. HIV-positive individuals are at risk of coinfection with HBV and HCV infections. The prevalence rates of coinfection with HBV and HCV in HIV-patients have been variable worldwide depending on the geographic regions, and the type of exposure.

**Aim:**

This study aimed to examine HBV and HCV coinfection serologically and determine the shared and significant factors in the coinfection of HIV-positive patients.

**Methods:**

This descriptive, cross-sectional study was carried out on 391 HIV-positive patients including 358 males and 33 females in Lorestan province, west Iran, to survey coinfection with HBsAg and anti-HCV. The retrospective demographic data of the subjects was collected and the patients' serums were analyzed by ELISA kits including HBsAg and anti-HCV. The collected data was analyzed with SPSS software (15) and Chi-square. Fisher's exact test with 5% error intervals was used to measure the correlation of variables and infection rates.

**Results:**

The results of the study indicated that the prevalence of coinfection in HIV-positive patients with hepatitis viruses was 94.4% (370 in 391), out of whom 57 (14.5%) cases were HBsAg positive, 282 (72%) cases were anti-HCV positive, and 31 (7.9%) cases were both HBsAg and anti-HCV positive.

**Conclusion:**

There was a significant correlation between coinfection with HCV and HBV and/or both among HIV-positive patients depending on different variables including sex, age, occupation, marital status, exposure to risk factors.(p < 0.001).

## Background

Human immunodeficiency virus (HIV), hepatitis B virus (HBV), and hepatitis C virus (HCV) are major public health concerns. Because of shared routes of transmission, HIV-HCV coinfection and HIV-HBV coinfection and/or both are common [1,2]. HIV-positive individuals are at risk of coinfection with HBV and HCV and/or both infections [3]. Coinfections of HBV and HCV with HIV have been associated with reduced survival, with an increased risk of progression to severe liver diseases and an increased risk of hepatotoxicity associated with antiretroviral therapy [1]. Worldwide, HIV is responsible for 38.6 million infections as estimated at the end of 2005 while HBV and HCV account for around 400 million and 170 million chronic infections, respectively. Moreover, among the HIV infected patients, 2-4 million are estimated to have chronic HBV coinfection while 4-5 million are coinfected with HCV [4]. An estimated one-third of the deaths in HIV patients are directly or indirectly related to liver diseases [5]. The prevalence rates of coinfection with HBV and HCV in HIV patients have been variable worldwide depending on the geographic regions, risk groups and the type of exposure involved which may be different not only from country to country, but also in different regions of the same country [6-8]. This study aimed to examine HBV and HCV coinfection in HIV-positive patients in Lorestan province, west Iran, serologically in order to recognize the prevalence rates of coinfection with these viruses in HIV-positive patients and the involving factors so that the results could increase clinical information in order to assess and treat the infections.

## Methods

This study aimed to examine HBV and HCV coinfection in HIV-positive patients in Lorestan province in Iran serologically in order to recognize the prevalence rates of coinfection with these viruses in HIV-positive patients and the involving factors so that the results could increase clinical information in order to assess and treat the infections.

### Study population

This descriptive, cross-sectional study from January 2007 to January 2008 was carried out on 391 HIV-positive patients including 358 males and 33 females in Lorestan province which is located in west Iran.

### Sampling

In this study, the serum samples from confirmed HIV-positive patients were measured by commercially available Enzyme Linked Immunosorbent Assay (ELISA) kits for the presence of HBsAg (Dialups, USA) and anti-HCV antibodies (Dialups USA, 3^rd ^generation).

### Statistical analysis

The retrospective demographic data of the subjects was collected and then the data was analyzed using the SPSS software -15.0 version - and Chi-square and Fisher's exact test with 5% level of significance was used to measure the association between the variables and infection rates.

## Results

The retrospective demographic data of the subjects showed that out of the 391 HIV-positive patients, 358 (91.6%) and 33 (8.4%) were males and females, respectively. Overall, the prevalence rates of coinfection of HBsAg and anti-HCV antibodies and both HBsAg and anti-HCV in HIV-positive patients were 282 (72%), 57 (14.5%) and 31(7.9%), respectively. (Table 1) The rate of the total HBsAg coinfection was 14.5% (57 in 391) in HIV-positive patients. Among the males, HIV/HBV coinfection was seen in 40 (11.2%) out of the 391 patients while among the females, HIV/HBV coinfection was observed in 17 (4.3%) out of the 391 patients. The rate of the total HCV coinfection was 72% (282 in 391) in HIV-positive patients. Among the males, HIV/HCV coinfection was seen in 274 (70%) out of the 391 patients while among the females, HIV/HCV coinfection was found in 8 (2%) out of the 391 patients. The rate of both HBV/HCV coinfections was 7.9% (31 in 391) in HIV-positive patients. Among the male HIV-positive patients, HBV/HCV coinfections were seen in 24 (6.1%) out of 391 while among the female HIV-positive patients, HBV/HCV coinfections were found in 7 (1.7%) out of 391. In addition, the following results from chi-square tests were obtained by investigating various variables including age, sex, occupation, marital status, and exposure to risk factors in HIV-positive patients:

**Table 1:** Prevalence rate of HBV & HCV positivity among HIV^+ ^patient

		HBsAg^+^		Anti-HCV^+^		Double Positive	
				
Variable	N (Percent)	N	P	N	P	N	P
**Total**	**391(100%)**	**57(14.5%)**		**282 (72%)**		**31 (7.9%)**	

**Sex**			<0.001		<0.001		<0.001
Male	358 (91.6%)	40(11.2%)		274(76.5%)		24 (6.7%)	
Female	33 (8.4%)	17(51.5%)		8 (24.3%)		7 (21.2%)	
**Age**			0.013		<0.001		<0.001
≤ 30	140 (35.8%)	18(12.9%)		108 (77.2%)		8 (5.7%)	
31-50	204 (52.1%)	27(13.2%)		151 (74%)		18 (8.9%)	
≥ 50	47 (12%)	12(25.5%)		23 (48.9%)		5 (10.6%)	
**Marital status**			<0.001		<0.001		<0.001
Single	195 (49.8%)	14(7.2%)		160 (82%)		11(5.7%)	
Married	166 (42.4%)	40(24%)		99 (59.7%)		18 (10.6%)	
Other	30 (7.6%)	3 (10%)		23 (76.7%)		2 (6.7%)	
**Risk Factor**			<0.001		<0.001		<0.001
IDU	202 (51.6%)	12 (5.9%)		172 (85.2%)		8 (3.1%)	
Sexual	11 (2.8%)	4 (3.4%)		3 (27.3%)		1 (9%)	
Transfusion	12 (3%)	0 (0%)		12 (100%)		0 (0%)	
Sex & IDU	56 (14.3%)	6 (10.7%)		36 (64.3%)		13(23.2%)	
Pregnant	5 (1.2%)	2 (40%)		0 (0%)		0 (0%)	
Other	105 (26.8%)	33(31.5%)		59 (56.2%)		9 (8.6%)	
**Occupation**			<0.001		<0.001		<0.001
Unemployed	221 (56.5%)	16 (7.2%)		174 (78.7%)		15 (6.8%)	
Self employed	63 (16.1%)	8 (12.7%)		47 (74.6%)		4 (6.3%)	
Worker	42)10.7%)	6 (14.3%)		33 (78.6%)		3 (7.2%)	
Employee	3 (0.7%)	1) 33.3%)		2 (66.7%)		0) 0%)	
housewife	32 (8.1%)	16 (50%)		8) 25%)		7 (21.9%)	
Farmer	4 (1%)	4 (100%)		0 (0%)		0 (0%)	
Student	12 (3%)	3 (25%)		9 (75%)		0 (0%)	
Driver	10 (2.5%)	2 (20%)		6 (60%)		20 (20%)	
Other	4 (1%)	1 (25%)		3 (75%)		0 (0%)	

HBsAg: HBV surface antigen, anti-HCV: antibody against HCV, N: number of patients, p: p-value, IDU: Injection drugs users

### Age

There was a significant relationship between age and condition of infection with HBV and HCV in HIV-positive patients (p = 0.0013) so that the infection rate with only HCV in HIV-positive patients under 40 was clearly more than those in other age groups (77.2% in patients under 30 and 74.2% in patients between 31-50), but for HIV it was less than the rates in other age groups (12.9% in patients under 30 and 13.2% in 31-50 age range), and finally HBV/HCV coinfections rate in the age groups of HIV-positive patients under 40 and above 60 was less than the rates in other groups (5.7% in patients under 30, and 8.9% in the patients of 31-50).

### Sex

Regarding gender, there was a significant relationship (p < 0.001), namely the infection with only HCV in HIV-positive men was more than the infection in HIV-positive women (69% and 24.3%, respectively) and the infection with HBV in HIV-positive female patients was more than that in HIV-positive male patients (51.5% vs. 11.2%), and finally HBV/HCV coinfections in HIV-positive women exceeded that for HIV-positive male patients (21.2% and 6.7%, respectively).

### Occupation

A significant relationship was found between occupation of HBV/HCV coinfections of HIV-positive patients (p < 0.001) so that the most infection with only HCV was in unemployed patients (78.7%) while farmers and ranchers (100%) and housewives (50%) suffered from the most infection with only HBV, and the most HBV/HCV coinfection was for housewives (21.9%).

### Marital status

Marital status was found to be a significant factor (p < 0.001) so that the infection with only HCV in single HIV-positive patients was more than that in married HIV-positive patients (82% and 59.7%, respectively). Moreover, the infection with only HBV in married HIV-positive patients was more than that in single HIV-positive patients (24% vs. 7.2%), and HBV/HCV coinfections rate in married HIV-positive patients was more than that for single HIV-positive patients (10.6% vs. 5.7%).

### Risk factors

A significant relationship was found between type of exposure to risk factors and condition of infection of HCV and HBV (p < 0.001), namely the rates of infection with only HCV in patients with blood transfusion and addiction to drug injection history being 100% and 85.2%, respectively were precisely more than those for other risk factors while the infection rate with only HBV in pregnancy (40%) and in having infected family member(s) (31.5%) was more than those for other factors. Moreover, and HBV/HCV coinfections rates in patients with suspicious sexual relationships and addiction to drug injection (Sex & IDU) were more than those for other risk factors (23.2%).

## Discussion

The primary purpose of the present study was to estimate the prevalence rate of HBV/HCV co-infection in HIV-positive patients in Lorestan province in Iran. Therefore, the study considered the common belief that most of HBV/HCV coinfections in HIV-positive patients are due to the shared routes of transmission of the viruses. So, the study showed some similarities and differences in the prevalence rates of HBV/HCV co-infection in HIV-positive patients in comparison to the studies carried out in our country, Iran, and in other developing and developed countries. They could be attributed to the epidemiologic conditions of the viruses that depend on various factors including the overlapping degree of risk factors in order to get infected with these viruses. In the US and Europe, HIV/HBV co-infection was reported to be 6 to 14% while reports for HIV/HCV varied in the range of 25 to 50% [9,10]. In a similar study carried out in Ahvaz - South Iran, the co-infection rates of HBV, HCV, and HBV/HCV in HIV-positive patients were found to be 44, 74, and 20%, respectively [11], and in our study the rates were 14.5% for HIV-HBV, 72% for HIV-HCV, and 7.9% for HBV/HCV coinfections. In another study performed on 150 HIV-positive patients in a private clinic in Tehran - the capital of Iran, HBV and HCV coinfection rates were 9.4 and 68%, respectively [12]. The results of a study in Nigeria conducted on 1779 HIV-positive patients revealed that the rates for HBsAg, HCV, and HBV/HCV coinfections were 11.9, 4.8, and 1%, respectively [13]. Moreover, in India, a study showed that the prevalence rate of HBsAg in HIV-positive patients was 3.4% while the rate for HCV-Ab was reported to be 0% [14]. Or in a similar study in northern India on 620 HIV-positive patients, the rate for HBV was 2.25%, for HCV 1.6%, and for both HBV/HCV coinfections it was less than 1% [15]. In Brazil the results of a study showed the rates of 6.4 and 5% for HBsAb and HCV-Ab coinfection in HIV-positive patients [16]. Also various reports of the prevalence rates of these viruses in HIV-positive patients exist [17-19]. The higher prevalence rate of HCV in HIV-positive patients in comparison to the rate for HBV in HIV-positive patients could be considered as noticeable and it could be attributed to diverse factors particularly lack of vaccines for HCV contrary to the existence of vaccines for HBV. Also, sexual transmission of this virus is lower in comparison to HBV and it is transmitted mostly via injection (especially in drug addiction) mostly because of the increasing rate of addiction in Iran [20]. In a study in Tabriz -west Iran, that investigated HIV/HBV/HCV co-infections in pregnant women, the prevalence rates of HBsAg, HCV-Ab, and HIV on 680 blood samples were 2.5, 1, and 0%, respectively [21]. Or in another study investigating the three viruses of HIV, HBV, and HCV on 2167 blood samples taken from blood donors, the rates for HBV, HCV, and HIV were 4.6%, 2.9%, and 0. 6%, respectively [22]. Many studies have been conducted in this realm [23], all showing high rates for the viruses in HIV-positive patients due to the above-mentioned factors. Some patients are currently infected with the three viruses of HIV, HBV, and HCV due to their shared risk factors. Consequently, coinfection with the three viruses will increase the risk of cirrhosis, liver deficiency, and mortalities in comparison to when a person is infected with only one of these viruses. Therefore, diagnosing HBV and HCV in HIV-positive patients is vital in order to take care of them and allot resources in health plans so that all HIV-positive patients have to be tested for both HVB and HCV [24,25].

## Abbreviations

HBV: Hepatitis B Virus; HCV: Hepatitis C Virus; HIV: Human Immunodeficiency Virus; ELISA: Enzyme-Linked Immunosorbent Assay; HBsAg: Hepatitis B surface antigen; Anti-HCV: Antibody Hepatitis C Virus.

## Competing interests

The authors declare that they have no competing interests.

## Authors' contributions

MM and GHT participated in the design and conducted the majority of the experiments in the study and helped to draft the manuscript, ASH contributed to the interpretation of the findings and revised the manuscript. EGH obtained and organized the clinical samples from HIV positive, FE performed analyses of data, HB carried out ELISA test, and YP performed wrote and editing the manuscript. All authors read and approved the final manuscript.
